# Interaction between caveolin-1 polymorphism and dietary fat quality indexes on visceral adiposity index (VAI) and body adiposity index (BAI) among overweight and obese women: a cross-sectional study

**DOI:** 10.1186/s12920-022-01415-5

**Published:** 2022-12-14

**Authors:** Rasool Ghaffarian-Ensaf, Farideh Shiraseb, Atieh Mirzababaei, Cain C. T. Clark, Khadijeh Mirzaei

**Affiliations:** 1 Department of Nutrition, Science and Research Branch, Islamic Azad University, Tehran, Iran; 2 Department of Community Nutrition, School of Nutritional Sciences and Dietetics, Tehran University of Medical Sciences (TUMS), P.O. Box, Tehran, 14155-6117 Iran; 3 Centre for Intelligent Healthcare, Coventry University, Coventry, CV1 5FB UK; 4 Food Microbiology Research Center, Tehran University of Medical Sciences, Teharn, Iran

**Keywords:** Caveolin-1 polymorphism, Visceral adiposity index, Body adiposity index, Dietary fat quality indexes

## Abstract

**Background and aims:**

Caveolin-1 (CAV-1) in adipocyte tissue and other body parts possesses numerous biological functions. In the present study, we sought to investigate the interaction between CAV-1 polymorphism and dietary fat quality indexes on visceral adiposity index (VAI) and body adiposity index (BAI) among overweight and obese women.

**Methods:**

This study was conducted on 386 women aged 18–48 years old. Biochemical measurements were assessed by standard protocols. We used a food frequency questionnaire (FFQ) to calculate the dietary intake and the indexes of dietary fat quality intake. Anthropometric values and body composition were measured by standard methods. Finally, the CAV-1 genotype was measured using the PCR–RFLP method.

**Results:**

We found marginally significant differences between AA and GG genotypes of waist-to-hip ratio (WHR) (*P* = 0.06) and BAI (*P* = 0.06) of participants after adjusting for potential confounders. For dietary intakes, after adjusting with the energy intake, mean differences in biotin (*P* = 0.04) and total fiber (*P* = 0.06) were significant and marginally significant, respectively. The interaction between two risk alleles (AA) with omega-6 to omega-3 ratio (W6/W3) on BAI, after adjustment for potential confounders (age, physical activity, energy intake, education), was marginally positive (β = 14.08, 95% CI = − 18.65, 46.81, *P* = 0.07). In comparison to the reference group (GG), there was a positive interaction between the two risk alleles (AA) with W6/W3 ratio on VAI (β = 2.81, 95% CI = 1.20, 8.84, *P* = 0.06) in the adjusted model.

**Conclusions:**

We found that there might be an interaction between CAV-1 genotypes with dietary quality fat indexes on VAI and BAI among overweight and obese women.

## Introduction

Overweight and obesity represent notable risk factors for many non-communicable diseases, and contribute significantly to global mortality rates [1–3]. Indeed, it has been estimated that by 2030, obesity and overweight will dramatically increase [4]. Obesity and overweight are associated with many diseases and clinical conditions, such as diabetes, cardiovascular disease (CVD), disability, depression, and early mortality [5].

Various anthropometric indexes can be used to get an overview of the body composition and health status of the body [6]. For example, body mass index (BMI), hip circumference (HC), waist circumference (WC), and WHR can be used to measure and monitor obesity [7]. In recent years, novel anthropometric indexes such as VAI and BAI have been introduced to measure body fat distribution accurately [8]. BAI appears to represent a good assessment of fat percentage using HC, height, and gender [9]. Whilst VAI is used to evaluate adipose tissue and its function based on gender by using anthropometric and biochemical parameters, such as waist circumference, body mass index, blood triglyceride concentration, and blood cholesterol [10].

Throughout the body, including adipose tissue, the plasma membrane contains a small (50–100 nm) complex, invaginations, and flask-shaped specialized structures, called caveolae, that have cholesterol and sphingolipids in their structure [11]. Caveolae are involved in lipid metabolism, signal transduction, and endocytosis [12–15]. Caveolae contain proteins called caveolin [16], which are integral membrane proteins that have scaffolding functions, and are regulators of many signaling pathways [17, 18]. There are three forms of caveolin: caveolin 1, caveolin 2, and caveolin 3, which are expressed differently and can all affect obesity [19]. Caveolin-1 is expressed in adipose tissue, caveolin-2 is expressed with caveolin-1 to form hetero-oligomers, and caveolin-3 is the muscle-specific isoform of caveolin [12, 15]. As an interactive gene, caveolin interacts with cholesterol and plays an essential role in its metabolism and regulation [20]. Due to the notable interaction of caveolin with cholesterol, animal studies have shown the significant effect of a high-cholesterol diet on the expression of the caveolin gene [21].

The quality of dietary fat intake is related to obesity and body fat percentage, and to examine this relationship, all aspects of fatty acids in the diet should be considered [22]. Indeed, most studies have been performed on the role of diet in the expression of the caveolin gene in animals. According to such studies, caveolin can be influenced and regulated by obesity and diet [23]. Fat consumption is known to impact adipose tissue [24]. Moreover, to evaluate the quality of dietary fat, several indicators can be used, including the cholesterol-saturated fat index (CSI) [25] and W6/W3 ratio [26].

This study sought to investigate the interaction between the quality of dietary fat intake and the CAV-1 gene and their relationship with visceral adiposity index and body adiposity index.

## Methods and materials

### Participants

Three hundred eighty-six women aged 18–48 years were randomly selected from among participants in a cross-sectional study performed in 2016–2017. The inclusion criteria were: obese or overweight (BMI > 25 kg/m^2^), no alcohol consumption, and no smoking. Women with CVD, kidney failure, stroke, thyroid disease, liver disease, cancer, inflammatory illnesses, and those taking any therapeutic medications and weight loss supplements, or any supplements that affect weight were excluded from the study. Pregnant and menopausal women and those who reported a total daily energy intake outside 800–4200 kcal (3344–17,556 kJ) were also excluded from the study. The study protocol was approved by the ethics committee of Tehran University of Medical Sciences (TUMS) with the following identification: IR.TUMS.VCR.REC.1397.920. All methods were carried out by relevant guidelines. All participants of the study completed a written informed consent.

### Measurement of biochemical parameters

All blood samples were collected at the Nutrition and Biochemistry laboratory of the School of Nutritional Sciences and Dietetics, TUMS. Serum triglycerides (TG) concentrations were assayed with triacylglycerol kits (Pars Azmoon Inc, Tehran, Iran) by using enzymatic colorimetric tests with Glycerol-3-phosphate oxidase Phenol 4-Aminoantipyrine Peroxidase (GPO-PAP). Total cholesterol (total-chol) levels were measured by the cholesterol oxidase Phenol 4-Aminoantipyrine Peroxidase (CHOD-PAP), and low-density lipoprotein (LDL) and high-density lipoprotein (HDL) were measured by the direct method and immune inhibition.

### Assessment of anthropometric measures

Weight was measured with digital scales and recorded to the nearest 100 g while the subjects were minimally clothed and unshod. Height was measured using tape while the subjects were standing, unshod, and had shoulders in a normal position. BMI was calculated by dividing the weight by the square of the height, waist circumference (WC) was measured at the narrowest part of the abdomen, HC was measured as the maximum circumference over the buttocks, and the minimal circumference was recorded to the nearest 0.1 cm. To reduce error, all measurements were taken by the same technician. For measuring the fat distribution, we used BAI and VAI (for women) with the following formulas:

- BAI was calculated using the following formula [9]:
  $${\text{BAI }} = \frac{{{\text{Hip }}\;{\text{Circumference}}\;\left( {{\text{cm}}} \right)}}{{height \left( m \right)^{1.5} }} - 18$$
- VAI was calculated using the formula [10]:
  $${\text{Women:}}\;{\text{VAI }} = \left( {\frac{{{\text{Waist}}\;{\text{Circumference}}\;{ }\left( {{\text{cm}}} \right)}}{{36.58 + \left( {1.89 \times BMI} \right)}}} \right) \times \left( {\frac{TG}{{0.81}}} \right) \times \left( {\frac{1.52}{{HDL}}} \right)$$

### Assessment of dietary intake

To evaluate participants’ dietary intake, we used a 147-item semi-quantitative FFQ, with high validity and reliability [27], during face-to-face interviews. The extracted FFQ values were then changed to grams/day. For the evaluation of macro-and micronutrient content, N4 software was used.

### Measurement of fat quality indexes

Fat quality indexes include CSI and W6/W3 ratio which are calculated through their respective formulas.

- CSI: Indicates the state of cholesterol and saturated fats, which helps a person in self-care against the state of cholesterol [25].
  $${\text{CSI}} = \frac{Cholesterol}{{Saturated\;fat}}$$
- w–6/w–3 ratio: Omega 6/Omega 3 are two essential fats that are categorized as PUFAs. The total amount of omega-6 s and omega-3 s are divided by each other to get a ratio [26].
  $$W{-}6/W{-}3\; Ratio = \frac{{\sum {\upomega }{-}6{ }}}{{\sum {\upomega }{-}3}}$$

### Genotyping

For genotyping the Cav-1 polymorphisms, DNA was extracted from whole blood via a Mini Columns kit (Type G; Genall; Exgene). The polymerase chain reaction-restriction fragment length polymorphism (PCRRFLP) technique was employed to investigate Cav-1 polymorphisms (rs3807992) in gene fragments (major allele G and minor allele A). PCR was carried out using the following primers:

F:3′AGTATTGACCTGATTTGCCATG5′R:5′GTCTTCTGGAAAAAGCACATGA-3′, according to pervious study [28]. We gave risk alleles GG, AG, and AA the values 2, 1, and 0.

### Assessment of other variables

We assessed the participants’ physical activity with a validated International Physical Activity Questionnaire (IPAQ) [29]. We also designed and used a standard socio-economic demographic questionnaire to collect general background information, including education and marital status.

### Statistical analysis

Statistical analysis was performed using SPSS v.25 software (SPSS Inc., IL, USA) and the significance level was, a priori, considered *P* ≤ 0.05, while *P* = 0.06 was considered as marginally significant. There were no missing values in the analysis. Our data followed a normal distribution, which was confirmed using the Kolmogorov–Smirnov test. All continuous data were expressed as means and standard deviation (SD), and categorical variables were expressed as numbers and percentages. The Pearson’s chi-square test was used for categorical variables and to determine the Hardy–Weinberg Equilibrium. One-way analysis of variance (ANOVA) was used to evaluate the association between dietary intake indexes, anthropometric parameters, and biochemical parameters, and analysis of covariance (ANCOVA) was used to evaluate and control for confounders. BMI consider as collinear variable for anthropometrics and body composition variables, these variables control with energy intake, age, and physical activity. To investigate the mean differences between groups, post-hoc multiple comparison analysis (Bonferroni corrected), was used. To estimate interactions between CAV-1 genotypes and VAI and BAI, we used a generalized linear model, conducting a crude and adjusted model, where we controlled several potential confounders (age, energy intake, physical activity, education). GG allele carriers were consider as the reference group.

## Result

### Study population characteristics

A total of 386 overweight and obese women were evaluated in this study. The participant characteristics were: age 36.67 (9.10) years, BMI 31.26 (4.29) kg/m^2^, VAI 2.48 (2.13), BAI 29.58 (7.62), CSI 9.74 (3.43), and W6/W3 ratio 12.65 (0.10). Among the participants, 286 (70.8%) women were married, and 110 (27.2%) had a good economic status. The overall prevalence of CAV-1 genotypes in the study was 50% (193), 23.3% (90), and 25.5% (103) for AA, AG, and AA, respectively.

### Baseline characteristics of study participants among the CAV-1 genotypes

The baseline characteristics of study participants, categorized according to the CAV-1 genotypes, are presented in Table 1. As shown in this table, *P* values for all variables were reported before the adjustment in the crude model, and after adjustment with potential confounders, including age, physical activity, energy intake, and BMI. BMI was considered as collinear for anthropometrics and body composition variables. In the crude model, there was a significant mean difference among the study participants in terms of BMI (*P* = 0.01), diastolic blood pressure (DBP) (*P* = 0.02), HDL (*P* = 0.001), LDL (*P* = 0.03), and VAI (*P* = 0.006). After adjustment for potential confounders, the mean difference became marginally significant in the WHR and BAI of participants. The mean difference of HDL and VAI remained significant in the adjusted model. According to Bonferroni post-hoc testing, the mean difference in WHR, VAI, and HDL was between AA and GG genotype groups, such that the mean was higher in AG genotype for WHR and VAI and was higher in GG genotype for HDL, while, in BAI, there was a difference between AA and AG genotypes, such that in the AG genotype was higher. In categorical variables, a significant mean difference among the participants was seen regarding their education status after controlling for cofounders. There was no significant difference in terms of other variables in Table 1.

**Table 1:** Baseline characteristics of study participants categorized according to the Cav-1 genotypes in obese and overweight women (n = 386)

Variables	CAV-1 genotypes				
	GG (N = 193)	AG (N = 90)	AA (N = 101)	*P* value	*P* value*
	Mean ± SD				
Demographic characteristics					
Age (year)	37.56 ± 9.49	35.85 ± 8.91	35.67 ± 8.71	0.15	0.33
Onset of obesity (year)	23.04 ± 10.02	23.76 ± 9.04	22.59 ± 8.41	0.72	0.19
Physical activity (MET/h)	1215.46 ± 2033.81	1379.12 ± 2823.33	1073.74 ± 1761.48	0.74	0.77
Anthropometric measurements					
Weight (kg)	79.71 ± 10.91	81.10 ± 12.24	83.01 ± 14.05	0.08	0.38
Height (cm)	161.30 ± 6.08	161.27 ± 5.34	160.68 ± 5.80	0.66	0.80
WC (cm)	96.61 ± 15.20	95.57 ± 18.23	100.26 ± 12.01	0.16	0.25
HC (cm)	113.59 ± 9.49	111.44 ± 6.76	116.10 ± 11.07	0.09	0.07
BMI (kg/m^2^)	30.68 ± 4.02	31.06 ± 4.06	32.19 ± 4.75	**0.01**	0.07
WHR	0.93 ± 0.05^b^	1.94 ± 9.59^b^	0.94 ± 0.05	0.19	**0.06**
BAI	29.35 ± 7.94	28.57 ± 8.72^a^	30.96 ± 5.80^a^	0.18	**0.06**
VAI	2.13 ± 1.24 ^b^	3.27 ± 3.47^b^	2.62 ± 2.05	**0.006**	**0.006**
Blood pressure					
SBP (mm Hg)	109.60 ± 15.05	113.05 ± 17.98	112.79 ± 12.04	0.19	0.78
DBP (mm Hg)	75.87 ± 10.77	79.45 ± 11.63	79.22 ± 8.85	**0.02**	0.47
Biochemical variables					
FBS (mg/dL)	87.98 ± 9.62	87 ± 11.39	86.91 ± 8.46	0.70	0.74
TC (mg/dL)	186.76 ± 33.74	187.58 ± 44.95	179.21 ± 30.67	0.29	0.27
TG (mg/dL)	113.11 ± 51.20	126.77 ± 61.42	123.90 ± 70.50	0.28	0.21
HDL (mg/dL)	49.07 ± 11.16^b^	42.84 ± 11.39^b^	44.90 ± 9.17	**0.001**	**0.03**
LDL (mg/dL)	98.88 ± 22.66	89.49 ± 26.45	92.54 ± 24.13	**0.03**	0.08
GOT (U/L)	18.27 ± 7.44	17.96 ± 7.03	17.95 ± 9.13	0.95	0.53
GPT (U/L)	19.04 ± 13.95	19.35 ± 11.66	20.71 ± 15.81	0.71	0.61
Supplements intake				0.18	0.83
Yes	87 (57.6%)	27 (17.9%)	37 (24.5%)		
No	83 (47.7%)	42 (24.1%)	49 (28.2%)		
Marital status				0.98	0.20
Single	55 (51.4)	24 (22.4)	28 (26.2)		
Married	137 (50.6)	63 (23.2)	71 (26.2)		
Education status				0.17	**0.009**
Illiterate	1 (25)	2 (50)	1 (25)		
Under diploma	20 (41.7)	12 (25)	16 (33.3)		
Diploma	68 (46.6)	33 (22.6)	45 (30.8)		
Bachelor and higher than	103 (57.2)	40 (22.2)	37 (20.6)		
Economic status				0.20	0.23
Poor	39 (45.3)	19 (22.1)	28 (32.6)		
Moderate	83 (47.4)	46 (26.3)	46 (26.3)		
Good	62 (59)	19 (18.1)	24 (22.9)		
Family history of obesity				0.55	0.15
Yes	129 (51.2)	56 (22.2)	67 (26.6)		
No	48 (45.3)	28 (26.4)	30 (28.3)		

*SD* standard deviation, *IPAQ* International Physical Activity Questionnaire, *WC* waist circumference, *BAI* body adiposity index, *VAI* visceral adiposity index, *SBP* systolic blood pressure, *DBP* diastolic blood pressure, *TG* triglyceride, *HDL* high density lipoprotein, *LDL* low density lipoprotein, *GOT* glutamic oxaloacetic transaminase, *GPT* glutamate pyruvate transaminase, *BMI* body mass index, *HC* hip circumference, *WHR* waist-to-hip ratio, *TC* total cholesterol

Values are represented as means ± SD. BMI is collinear

Categorical variables: N (%)

Independent T test (*P* value) was performed to identify significant differences between Cav-1 genotypes in crude model

ANCOVA (*P* value*) was performed to adjusted potential confounding factors (age, energy intake, Physical activity, BMI)

BMI consider as collinear variable for anthropometrics and body composition variables, these variables control with energy intake, age and, physical activity

Bold indicate *p* values < 0.05 were considered as significant, and *p* values = 0.06 were considered as marginally significant

^a^The significant difference was seen between AA and AG

^b^The significant difference was seen between GG and AG

### Dietary intakes of all subjects according to CAV-1 genotypes in obese and overweight women

Dietary intakes of all subjects according to CAV-1 genotypes in obese and overweight women are presented in Table 2. Regarding fat components of foods, no significant difference was found.in crude and adjusted models. The result was the same for macronutrients and energy, with no significant difference in the crude and adjusted models. According to micronutrients, there was no significant difference among participants in the crude model. But after adjustment for energy intake, mean differences in Biotin (*P* = 0.04) and total fiber (*P* = 0.06) were significant and marginally significant, respectively. There was no significant difference in terms of other variables in Table 2.

**Table 2:** Dietary food intakes of all subjects according to Cav-1 genotypes in obese and overweight women (n = 386)

Variables	GG (n = 193)	AG (n = 90)	AA (n = 101)	*P* value	*P* value^a^
	Mean ± SD				
Fat components of foods					
Cholesterol (g/day)	259.32 ± 8.95	256.24 ± 13.73	242.17 ± 12.18	0.51	0.26
Saturated Fats (mg/day)	28.55 ± 0.94	28.19 ± 1.45	27.12 ± 1.28	0.66	0.23
MUFA (mg/day)	31.50 ± 0.95	31.35 ± 1.46	31.01 ± 1.30	0.95	0.73
PUFA (mg/day)	19.99 ± 0.72	20.14 ± 1.10	20.29 ± 0.98	0.96	0.99
Linoleic acid (mg/day)	17.28 ± 0.68	17.38 ± 1.05	17.53 ± 0.93	0.97	0.99
Linolenic acid (mg/day)	1.20 ± 0.05	1.30 ± 0.08	1.23 ± 0.07	0.60	0.62
EPA (mg/day)	0.03 ± 0.003	0.03 ± 0.005	0.03 ± 0.00	0.61	0.63
DHA (mg/day)	0.10 ± 0.01	0.10 ± 0.01	0.12 ± 0.01	0.56	0.59
Trans fats (mg/day)	0.001	0	0.001	0.08	0.07
CSI	9.49 ± 0.26	9.50 ± 0.40	9.36 ± 0.35	0.95	0.96
W6/W3	12.65 ± 0.009	12.64 ± 0.01	12.64 ± 0.01	0.95	0.17
Macronutrients and energy					
Energy (Kcal)	2621.64 ± 721.37	2597.22 ± 723.78	2677.85 ± 858.75	0.84	-
Carbohydrate (g/day)	370.78 ± 10.29	374.19 ± 15.79	379.33 ± 14.01	0.88	0.84
Protein (g/day)	88.49 ± 2.43	90.42 ± 3.73	88.32 ± 3.31	0.89	0.73
Total fat (g/day)	94.10 ± 2.71	95.89 ± 4.16	93.89 ± 3.69	0.92	0.77
Micronutrients					
Sodium (mg/day)	4200.47 ± 121.13	4406.56 ± 185.93	4237.80 ± 164.91	0.64	0.59
Potassium (mg/day)	4360.21 ± 131.69	4449.45 ± 202.13	4143.65 ± 179.28	0.44	0.07
Vitamin A (RAE) (mg/day)	801.97 ± 34.99	753.84 ± 53.71	764.69 ± 47.64	0.69	0.51
Beta carotene (mg/day)	5490.89 ± 303.02	5219.52 ± 465.11	4940.81 ± 412.53	0.55	0.45
Vitamin C (mg/day)	197.04 ± 10.81	194.27 ± 16.59	197.43 ± 14.71	0.98	0.94
Calcium (mg/day)	1161.14 ± 35.38	1184.05 ± 54.31	1156.44 ± 48.17	0.92	0.83
Iron (mcg/day)	18.65 ± 0.50	18.46 ± 0.77	19.02 ± 0.68	0.85	0.51
Vitamin D (ug/day)	2.01 ± 0.13	2.05 ± 0.21	1.92 ± 0.19	0.88	0.83
Vitamin E (mg/day)	17.61 ± 0.77	16.84 ± 1.19	17.63 ± 1.05	0.84	0.78
Thiamin (mg/day)	2.04 ± 0.05	2.08 ± 0.08	2.17 ± 0.07	0.37	0.12
Riboflavin (mg/day)	2.20 ± 0.06	2.21 ± 0.10	2.21 ± 0.09	0.99	0.95
Niacin (mg/d)	25.21 ± 0.78	25.24 ± 1.20	25.77 ± 1.06	0.90	0.89
Vitamin B6 (mg/day)	2.18 ± 1.06	2.22 ± 0.09	2.10 ± 0.08	0.60	0.16
Total Folate (µg/day)	603.19 ± 15.003	608.42 ± 23.02	621.79 ± 20.42	0.76	0.73
Vitamin B12 (mcg/day)	4.26 ± 0.20	4.37 ± 0.31	4.54 ± 028	0.72	0.78
Biotin (mcg/day)	39.70 ± 1.43	39.03 ± 2.20	35.26 ± 1.95	0.17	**0.04**
Pantothenic acid (mg/day)	6.56 ± 0.20	6.63 ± 0.31	6.28 ± 0.27	0.63	0.27
Vitamin K (mcg/day)	228.18 ± 16.61	201.62 ± 25.49	196.37 ± 22.61	0.45	0.41
Phosphor (mg/day)	1644.12 ± 43.89	1667.96 ± 67.37	1606.34 ± 59.76	0.78	0.30
Magnesium (mg/day)	464.57 ± 12.53	463.81 ± 19.23	445.40 ± 17.05	0.64	0.10
Zinc (mg/day)	12.94 ± 0.35	13.13 ± 0.54	12.75 ± 0.48	0.87	0.45
Copper (mcg/day)	2.01 ± 0.06	1.95 ± 0.09	2.03 ± 0.08	0.83	0.48
Manganese (mg/day)	7.02 ± 0.24	7.40 ± 0.36	6.92 ± 0.32	0.58	0.46
Selenium (mcg/day)	119.26 ± 3.61	121.68 ± 5.55	120.58 ± 4.92	0.93	0.96
Chromium (mg/day)	0.11 ± 0.007	0.11 ± 0.01	0.10 ± 0.01	0.77	0.69
Total fiber (g/day)	44.55 ± 1.59	43.17 ± 2.44	48.95 ± 2.17	0.15	**0.06**
Caffeine (mg/day)	145.83 ± 13.10	177.69 ± 20.12	141.93 ± 17.84	0.33	0.34

*SD* standard deviation, *MUFA* monounsaturated fatty acid, *PUFA* polyunsaturated fatty acid, *EPA* eicosapentaenoic acid, *DHA* docosahexaenoic acid, *CSI* cholesterol-saturated fat index

Values are represented as means ± SD

Independent T test (*P* value) was performed to identify significant differences between Cav-1 genotypes in crude model

ANCOVA (*P* value*) was performed to adjusted potential confounding factors (energy intake)

Bold indicate *p* values < 0.05 were considered as significant, and *p* values = 0.06 were considered as marginally significant

### The association of genotype variant of CAV-1 with BAI and VAI in crude and adjusted linear regression models in overweight and obese women

The association of the genotype variant of CAV-1 with BAI and VAI in the crude and adjusted linear regression model is presented in Table 3. After controlling for the potential confounders (age, energy intake, physical activity), in comparison to the reference group (GG), there was a marginally significant positive association between the AA genotype and BAI (β = 0.94, 95% CI = 0.006, 4.33, *P* = 0.06). A positive association between AG genotype and VAI, compared to the reference group (GG), was observed in the crude model (β = 67.77, 95% CI = 26.06, 109.48, *P* = 0.001). On the other hand, after adjusting for confounders (age, energy intake, physical activity), a positive association was found in comparison to the reference group (GG) for the AA and AG genotypes with VAI (β = 42.12, 95% CI = −2.74, 86.98, *P* = 0.06), (β = 84.12, 95% CI = 32.16, 136.07, *P* = 0.002), respectively.

**Table 3:** Association of genotype variant of Cav-1 with BAI and VAI in crude and adjusted linear regression models in overweight and obese women (n = 386)

Variables	Model	Genotypes	B	95% CI	*P* value
BAI	Crude	AA	1.60	− 0.61, 3.81	0.15
		AG	− 0.78	− 3.04, 1.47	0.49
	Adjusted	AA	0.94	0.006, 4.33	**0.06**
		AG	− 2.06	− 5.89, 1.76	0.29
VAI	Crude	AA	32.19	− 5.77, 70.16	0.09
		AG	67.77	26.06, 109.48	**0.001**
	Adjusted	AA	42.12	− 2.74, 86.98	**0.06**
		AG	84.12	32.16, 136.07	**0.002**

*CI* confidence interval, *BAI* body adiposity index, *VAI* visceral adiposity index

Comparisons between groups were determined based on logistic regression analysis

Generalized linear model Adjusted (age, energy intake, Physical activity, education)

Bold indicate *p* values < 0.05 were considered as significant, and *p* values = 0.06 were considered as marginally significant

GG consider as reference group

### Interactions between CAV-1 genotypes and CSI on BAI and VAI in overweight and obese women

**Fig. 1:**
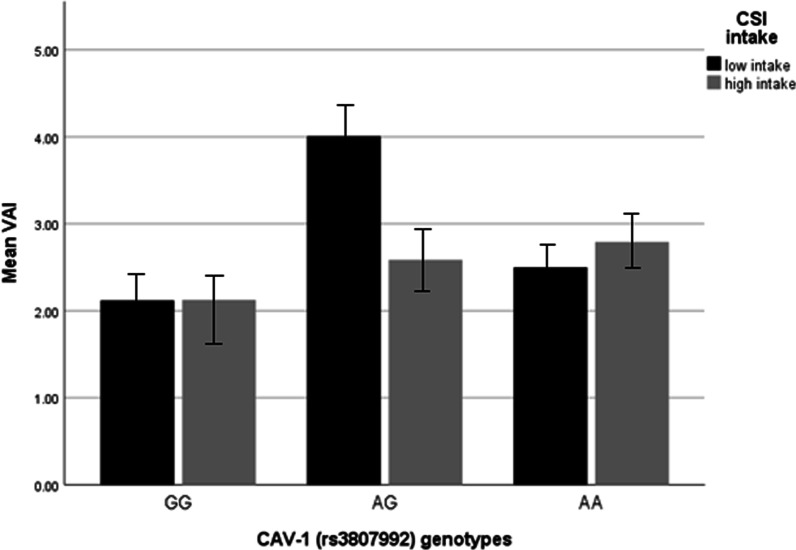
Interaction between CAV-1 genotypes (GG as the reference group) and CSI intake on VAI. VAI (The *P* value for AA genotype: 0.61; *P* value for CSI intake: 0.99; *P* value for interaction between AA genotype and CSI intake: 0.28). In this figure low and high intake of CSI is based on medians, low intake is considered as ≤ 9.405 and high intake is considered as ≥ 9.405

The interaction of CAV-1 genotypes with CSI on BAI and VAI in the crude and adjusted model is presented in Table 4. In the crude model, in terms of BAI, there was a negative interaction between two risk alleles (AG) with CSI (β = − 0.30, 95% CI = − 0.99, 0.37, *P* = 0.38) in comparison to the reference group (GG), and after controlling for potential confounders (age, physical activity, energy intake, education), this interaction remained negative between AG and CSI (β = −0.58, 95% CI = − 1.67, 0.50, *P* = 0.29). For VAI, a positive interaction was observed between two risk alleles (AA) with CSI in the crude and adjusted model, in comparison to reference group (GG) (β = 0.11, 95% CI = − 0.09, 0.31, *P* = 0.28), (β = 0.11, 95% CI = 0.11, 0.34, *P* = 0.04), respectively (Fig. 1).

**Table4:** Interactions between Cav-1 genotypes and dietary fat quality indexes in overweight and obese women (n = 386)

Interaction type	Β	95% CI	*P* value
*BAI*			
Crude			
AA*CSI	− 0.16	− 0.64, 0.61	0.96
AG*CSI	− 0.30	− 0.99, 0.37	0.38
Adjusted			
AA*CSI	− 0.36	− 1.41, 0.68	0.49
AG*CSI	− 0.58	− 1.67, 0.50	0.29
*BAI*			
Crude			
AA*W6/W3	4.03	− 22.02, 30.09	0.76
AG*W6/W3	− 6.45	− 37.63, 24.73	0.68
Adjusted			
AA*W6/W3	14.08	18.65, 46.81	0.07
AG*W6/W3	− 12.91	− 52.43, 26.61	0.52
*VAI*			
Crude			
AA*CSI	0.11	− 0.09, 0.31	0.28
AG*CSI	− 0.09	− 0.31, 0.12	0.37
Adjusted			
AA*CSI	0.11	0.11, 0.34	0.04
AG*CSI	− 0.06	− 0.32, 0.19	0.62
*VAI*			
Crude			
AA*W6/W3	2.04	− 4.01, 8.11	0.50
AG*W6/W3	− 3.76	− 10.81, 3.29	0.29
Adjusted			
AA*W6/W3	2.81	1.20, 8.84	0.06
AG*W6/W3	− 5.66	− 14.79, 3.46	0.22

*CI* confidence interval, *BAI* body adiposity index, *VAI* visceral adiposity index, *CSI* cholesterol-saturated fat index

*P* value with unadjusted (crude)

*P* value with adjustments for potential confounding factors including (age, physical activity, energy intake, education)

### Interactions between CAV-1 genotypes and W6/W3 ratio on BAI and VAI in overweight and obese women

**Fig. 2:**
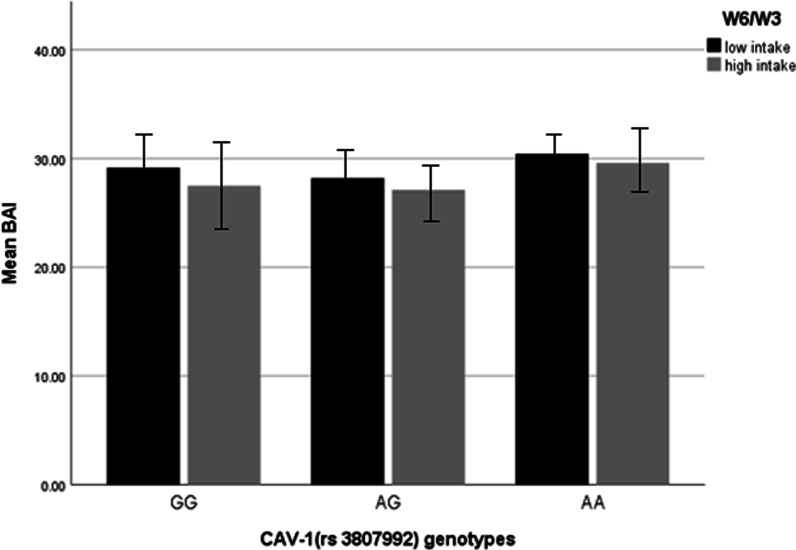
Interaction between CAV-1 genotypes (GG as the reference group) and W6/W3 intake on BAI. VAI (The *P* value for AA genotype: 0.76; *P* value for W6/W3 intake: 0.52; *P* value for interaction between AA genotype and W6/W3 intake: 0.76). In this figure low and high intake of W6/W3 is based on medians, low intake is considered as ≤ 12.646 and high intake is considered as ≥ 12.646

**Fig. 3:**
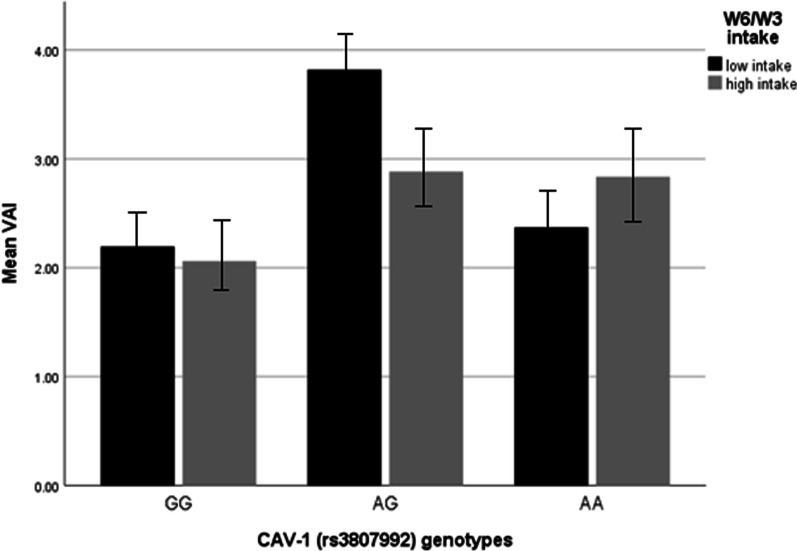
Interaction between CAV-1 genotypes (GG as the reference group) and W6/W3 intake on VAI. VAI (The *P* value for AA genotype: 0.51; *P* value for W6/W3 intake: 0.80; *P* value for interaction between AA genotype and W6/W3 intake: 0.50). In this figure low and high intake of W6/W3 is based on medians, low intake is considered as ≤ 12.646 and high intake is considered as ≥ 12.646

In the crude model, the interaction between two risk alleles (AG) with W6/W3 ratio on BAI, in comparison to the reference group (GG), was negative but not significant (β = − 6.45, 95% CI = −37.63, 24.73, *P* = 0.68), and the interaction between two risk alleles (AA) with W6/W3 ratio on BAI after adjustment for potential confounders (age, physical activity, energy intake, education) was marginally positive (β = 14.08, 95% CI = − 18.65, 46.81, *P* = 0.07) (Fig. 2). In terms of VAI, in comparison to the reference group (GG), there was a positive interaction between two risk alleles (AA) with W6/W3 ratio (β = 2.81, 95% CI = 1.20, 8.84, *P* = 0.06) in the adjusted model (Fig. 3).

## Discussion

To our knowledge, this cross-sectional study is the first to investigate the interaction between CAV-1 genotypes and dietary fat quality indexes on VAI and BAI among overweight and obese women. Accordingly, our results showed that there might be an interaction between CAV-1 genotypes and dietary fat quality indexes (CSI, W6/W3 ratio) on BAI and VAI in overweight and obese women.

We found that the interaction of increased adherence to W6/W3 ratio consumption with AA carriers of CAV-1 was positive on VAI and BAI. We also noted a positive interaction between CSI with AA allele carriers on VAI. To our knowledge, no study has been conducted directly on this subject, but according to some related studies, CAV-1 may directly impact the regulation of HDL, TG, and cholesterol, and in general, CAV-1 affects fatty acid metabolism [20, 30, 31]. In another study, the CAV-1 expression was decreased in the adipocytes of obese subjects [32]. An animal study showed that consumption of a high-cholesterol diet could affect the expression of CAV-1 [21]. We found that AA allele carriers of CAV-1 have higher BAI than other groups, while in terms of VAI, the AG genotype group was higher than others. BAI and VAI represent novel indicators of obesity that have been scarcely studied [33, 34]. Females with AA allele carriers had a significantly higher BMI compared to the reference group (GG), which is consistent with previous studies [28]. According to Catalan et al., CAV-1 expression in visceral adipose tissue and subcutaneous adipose tissue is positively associated with BMI and body fat [30].

According to our results, females with the AG allele carriers have higher WHR and DBP values than the GG group, while HDL and LDL values were higher in the reference group (GG). As previously mentioned, CAV-1 has a critical role in lipid homeostasis, which can affect adipose tissue, blood vessels, and the liver to alter the regulation of TG, cholesterol, VLDL, and HDL [31]. However, our results did not show any significant difference in the macronutrients, energy, and fat components of the foods of subjects, with the only difference found in biotin and total fiber. The subjects' varied intake of foods high in biotin or containing biotin is likely the cause of this difference.

Caveolae are formed from lipid rafts and contain cholesterol, glycosphingolipids, and CAV-1 [35], and can elicit an uptake of lipid metabolites, including several fatty acid species, triacylglycerol, and cholesterol [36–38]. The roles of CAV-1 are variegated, with its most essential functions in cholesterol homeostasis, signal transduction, cellular and systemic lipid metabolism, and regulation of lipid and lipoprotein metabolism [30, 31, 39]. CAV-1 is expressed in different body parts, such as fibroblasts, epithelial and endothelial cells, and adipocytes [40]. Interestingly, the CAV-1 works as an element in lipid droplets and can affect lipid droplet accumulation and breakdown [41]. According to Chang et al., a high-fat diet causes CAV-1 to be secreted more in adipose tissue than a normal diet in mice; therefore, adipose tissue may be considered the main source of CAV-1 secretion [42]. Fat consumption can change the effect of Cav-1 polymorphism function on obesity and metabolic syndrome [28]. For example, high SFA intake can increase the adverse effects of Cav-1 in terms of metabolic syndrome and obesity [28]. One study revealed that A allele carriers with a high intake of PUFA, had a lower risk of metabolic syndrome. Consumption of SFA and PUFA can change caveolae contents and cell signaling [43–45].

CAV-1 is involved in lipogenesis and adipogenic processes as CAV-1 mRNA [46]., and CAV-1 can functionally suppress transforming growth factor-beta (TGF-β) by modifying the phosphorylation state of SMAD., Another potential mechanism is that the interaction between CAV-1 with transforming growth factor-beta type 1 receptor (TαR-1) may influence TGF-β inhibition [47]. Further, CAV-1 can impact cytokine signaling by interactions with the Janus kinase (JAK) family, cytokine receptors, and the proteasome pathway [15, 48].

Several limitations in the present study must be considered when interpreting our findings. This study was cross-sectional, thereby precluding causal inferences from being drawn. Next, the sample size used to conduct this study was small, and we used an FFQ to investigate the intakes of subjects, which, given its’ self-reported nature, may be influenced by recall bias. Furthermore, this study was conducted only on women, so the results may not generalize to all sexes. Nevertheless, despite the noted limitations, our study has numerous strengths, including; this is, to our knowledge, the first study to have investigated the interaction between CAV-1 genotypes with dietary fat quality indexes on VAI and BAI among overweight and obese women, moreover; we used detailed genetic factors in the study. Further, since we only conducted this study on women, greater specificity and insight may be gleaned.

## Conclusion

Based on the present study’s finding, it appears that probably there may be an interaction between CAV-1 genotypes with dietary quality fat indexes and an association with VAI and BAI among overweight and obese women. Two risk alleles (AA) significantly interacted with CSI and W6/W3 ratio consumption and impacted BAI and VAI. In addition, we saw an association between the AG genotype and VAI. Nevertheless, we recommend that more studies be done in this area to confirm the integrity of our findings.

## Data Availability

The data that confirm the findings of this study are available from Khadijeh Mirzaei Data are available from the authors upon reasonable request and with permission of Khadijeh Mirzaei.

## Abbreviations

BAI: Body adiposity index
BMI: Body mass index
CAV-1: Caveolin-1
CI: Confidence interval
CSI: Cholesterol-saturated fat index
CVDs: Cardiovascular diseases
DBP: Diastolic blood pressure
DNA: Deoxyribonucleic acid
FFQ: Food frequency questionnaire
HC: Hip circumference
HDL: High-density lipoprotein
Il-1β: Interleukin 1 beta
IPAQ: International physical activity questionnaire
JAK: Janus kinase
LDL: Low-density lipoprotein
MET: Metabolic equivalent of task
mRNA: Messenger ribonucleic
PUFA: Polyunsaturated fatty acid
SD: Standard deviation
TG: Triglyceride
TGF-β: Transforming growth factor beta
TNF-α: Tumor necrosis factor alpha
TβR-1: Transforming growth factor-beta type 1 receptor
VAI: Visceral adiposity index
WC: Waist circumference
WHR: Waist-to-hip ratio
W6/W3: Omega-6 to omega-3 ratio
